# Multiple roles of Sonic Hedgehog in the developing human cortex are suggested by its widespread distribution

**DOI:** 10.1007/s00429-018-1621-5

**Published:** 2018-02-28

**Authors:** Fani Memi, Nada Zecevic, Nevena Radonjić

**Affiliations:** 1 Department of Neuroscience, University of Connecticut Health Center, Farmington, CT 06030 USA; 2 Department of Psychiatry, University of Connecticut Health Center, 263 Farmington Avenue, Farmington, CT 06030 USA; 3 Department of Cell and Developmental Biology, University College London, 21 University Street, London, WC1E 6DE UK

**Keywords:** Cerebral cortex, Human fetal brain, Morphogen, SHH receptors

## Abstract

Sonic Hedgehog (Shh) plays an instrumental role in brain development, fine-tuning processes such as cell proliferation, patterning, and fate specification. Although, mutations in the *SHH* pathway in humans are associated with various neurodevelopmental disorders, ranging from holoprosencephaly to schizophrenia, its expression pattern in the developing human brain is not well established. We now determined the previously not reported wide expression of *SHH* in the human fetal cerebral cortex during most of the gestation period (10–40 gestational weeks). This spatiotemporal distribution puts Shh in a position to influence the fundamental processes involved in corticogenesis. *SHH* expression increased during development, shifting from progenitor cells in the proliferative zones to neurons, both glutamatergic and GABAergic, and astrocytes in upper cortical compartments. Importantly, the expression of its downstream effectors and complementary receptors revealed evolutionary differences in *SHH*-pathway gene expression between humans and rodents.

**Electronic supplementary material:**

The online version of this article (10.1007/s00429-018-1621-5) contains supplementary material, which is available to authorized users.

## Introduction

Corticogenesis is a developmental process that requires coordination of the signaling pathways providing mitogenic signals and guiding the regulation of symmetric/asymmetric divisions of neuronal progenitors, positional information, differentiation signals, and a balance of excitation and inhibition in the cortex. Cortical development has been extensively studied in mice, and although it is considered to be generally well conserved in mammals, a number of changes and novelties have emerged during evolution that may underlie the biological basis for the higher cognitive and motor abilities that are specific to humans (Geschwind and Rakic 2013; Silbereis et al. 2016). Sonic Hedgehog (Shh) is a pleiotropic protein that plays a major role in most of the aforementioned processes during corticogenesis, including dorso-ventral patterning, the specification of interneurons and oligodendrocytes, as well as cortical circuitry formation (Fuccillo et al. 2006; Echelard et al. 1993; Ericson et al. 1995; Xu et al. 2005; Tekki-Kessaris et al. 2001; Harwell et al. 2012).

During mouse embryonic development, Shh is highly expressed in the ventral forebrain, where it plays a critical role in patterning during a specific developmental time window (embryonic days 9.5–12.5) (Xu et al. 2005; Fuccillo et al. 2004; Machold et al. 2003). Notably, changes in the concentration of Shh and the duration of Shh exposure influence the specification of different ventral progenitors and their neuronal progeny, which range from hypothalamic and striatal projection neurons to cortical and striatal interneurons (Maroof et al. 2013; Tyson et al. 2015). Despite the sparse expression of Shh in the mouse dorsal telencephalon (Dahmane et al. 2001), conditional inactivation of the Shh pathway leads to the defective proliferation of intermediate progenitor cells and to microcephaly (Komada et al. 2008). These results point to the concentration-dependent functions of this morphogen in both dorsal and ventral forebrain developments.

The significance of *SHH* in human brain development is illustrated by the dramatic consequences of *SHH*-pathway gene disruption, which include holoprosencephaly, seizure disorders, language or cognitive impairment, Down syndrome, hyperactivity, and schizophrenia (Heussler et al. 2002; Nanni et al. 1999; Belloni et al. 1996; Odent et al. 1999; Santiago et al. 2006; Currier et al. 2012; Betcheva et al. 2013). Many of these conditions are the result of *SHH* haploinsufficiency, thus highlighting the importance of *SHH* gene dosage in humans (Chiang et al. 1996). Although in human embryos (Carnegie stages 12–16), the expression of *SHH* has been demonstrated ventrally, in the notochord, in the floorplate of the spinal cord, and in the hindbrain (Odent et al. 1999; Hajihosseini et al. 1996), there is a lack of information regarding the sources of SHH in the developing cerebral cortex.

A prerequisite for understanding the physiological role of SHH signaling during cortical development is the determination of its distribution and identification of its cellular sources in the prenatal human cortex. Our initial results, obtained in vitro, showed that at mid-gestation (around 20 gestational weeks, gw), *SHH* is expressed by radial glia cells (RGCs) and that treatment with exogenous SHH favours the generation of Nkx2.1^+^ progenitors over calretinin (CalR^+^) cells, while it has no effect on the generation of pyramidal neurons (Radonjic et al. 2016). In the present study, we used in situ hybridization (ISH) to analyze the distribution of *SHH*-expressing cells in the prenatal human forebrain. We studied a wide spectrum of gestational ages (8–40 gw), using cryo-sections from throughout the rostro-caudal brain axis. We also combined fluorescence ISH (FISH) with cell-type-specific immunostaining and identified the cell types that express this morphogen. Finally, we analyzed, for the first time, the expression pattern of the known SHH receptors and downstream effectors in comparison with SHH expression in the early and late corticogenesis. These results contribute to the better understanding of cortical development and point to the importance of further studies of SHH signaling in neuropsychiatric disorders.

## Materials and methods

### Human fetal brain tissue

Human fetal brains (*n* = 30) from 8 to 40 gw (gestational weeks correspond to postconceptional weeks; full term = 40 gw) (Table S1) were obtained from the Human Fetal Tissue Repository at the Albert Einstein College of Medicine (Bronx), Advanced Bioscience Resources (Alameda, CA), StemEx (Diamond Springs, CA, USA) and the joint MRC/Wellcome Trust-funded Human Developmental Biology Resource (http://www.hdbr.org) after legal abortions with appropriate maternal written consent and approval from the Ethics Committees of the participating institutions. All human materials were handled with special care and following all necessary requirements and regulations set by the Ethics Committee of the University of Connecticut and the Helsinki Declaration. Fetal age was estimated on the basis of weeks after last period, crown-rump length, and anatomical landmarks. In all studied fetuses, ultrasound and gross neuropathological examination were used to exclude those with brain pathology. The dissected tissues were fixed in 4% formaldehyde solution in 0.1 M phosphate buffer, cryoprotected by immersion in 30% sucrose, embedded in Tissue Tek (Sakura), frozen, and preserved in − 80 °C until needed. The tissues used for in situ hybridization (ISH) and immunohistochemistry were cut into 15-µm-thick sections.

### In situ hybridization

The human full-coding sequences (CDS) for *GLI1, GLI2, GLI3*, and *PTCH 1* were purchased from Dharmacon. The human *SHH* CDS was a gift from Cliff Tabin (Marigo et al. 1995) (pBS hShh CT#401 Addgene # 13996). For the *SMO, BOC, GAS1*, and *CDON* probes, cDNAs from human fetal brain (18 gw) were used as templates. The riboprobes were generated from a PCR fragment containing the transcription promoter sites T3/T7/SP6 (see Table S2), by in vitro transcription using digoxigenin (DIG)-UTP (Roche) as the label. ISH was performed as previously described (Radonjic et al. 2014). Briefly, cryo-sections (15 µm) were dried at room temperature (RT) for 2 h, fixed for 10 min with 4% paraformaldehyde (PFA), and washed twice in diethyl pyrocarbonate (DEPC)-treated phosphate buffer solution (PBS) before overnight incubation at 70 °C in hybridization buffer containing 1 × DEPC-treated “salts” (200 mM NaCl, 5 mM EDTA, 10 mM Tris, pH 7.5, 5 mM NaH2PO4·2H2O, 5 mM Na2HPO4; Sigma-Aldrich), 50% deionized formamide (Roche), 0.1 mg/mL of RNase-free yeast tRNA (Invitrogen, Carlsbad, CA, USA), 1 × Denhardts (RNase/DNase-free; Invitrogen), 10% dextran sulfate (Sigma-Aldrich) containing 100–500 ngmL of digoxigenin (DIG)-labeled RNA probe. After hybridization, the sections were washed three times in a solution containing 50% formamide 1 × SSC (saline-sodium citrate, Invitrogen) and 0.1% Tween 20 (Sigma-Aldrich) at 65 °C, and twice at RT in 1 × MABT (20 mM maleic acid, 30 mM NaCl, 0.1% Tween 20; Sigma-Aldrich) before incubating in a solution containing 2% blocking reagent (Roche) and 10% heat-inactivated sheep serum in MABT, followed by overnight incubation in alkaline-phosphatase-conjugated anti-DIG antibody (1:1500; Roche). Fast Red (Roche) was used for the fluorescent colorimetric detection of probe (FISH) by incubation in 100 mM Tris, pH 8.2, 400 mM NaCl containing Fast Red for 1–2 h at 37 °C. Alternatively, the TSA (tyramide signal amplification) kit (Perkinelmer) was used for FISH, in combination with anti-DIG-POD antibody (1:500, Roche). The sections were counterstained with bis-benzimide and cover-slipped using Fluoromount G mounting medium. The specificity of the procedure was assessed with a probe corresponding to the sense strand of the respective genes.

### Immunohistochemistry after ISH

After ISH, sections were subjected to antigen retrieval in 0.1 M citric acid, pH 9.0, and blocked in 10% normal goat serum (NGS)/PBS containing 0.2% Triton (PBST). Following overnight incubation with primary antibody (see Table S3 for details), the sections were thoroughly washed in PBST and incubated with secondary Alexa 488- or Alexa 555-conjugated antibodies (Life Technologies) for 2 h at RT. Alternatively, an ABC kit was used followed by DAB development. Nuclei were counterstained for 5 min at RT with the nuclear stain bis-benzimide (Sigma).

### Quantification of immunolabeled cells

Immunolabeled sections were visualized with an Axioscope microscope (Zeiss) equipped with Axiovision software and photographed using a digital camera. Three samples (22–24 gw) were used for the quantifications presented in Figs. 4 and 5. Nuclear staining allowed the delineation of areas of interest (e.g., CP, SVZ, IZ, and GE). Ten images for each region of interest were observed at 40× magnification and the number of cells was counted using the Photoshop CS6 Count Tool. A descriptive analysis of the data was performed using the Excel Data analysis plug-in. The percentages of double-positive cells expressing SHH are presented together with the standard error of the mean (SEM).

## Results

### Distribution of *SHH* mRNA in the developing human forebrain

**Fig. 1:**
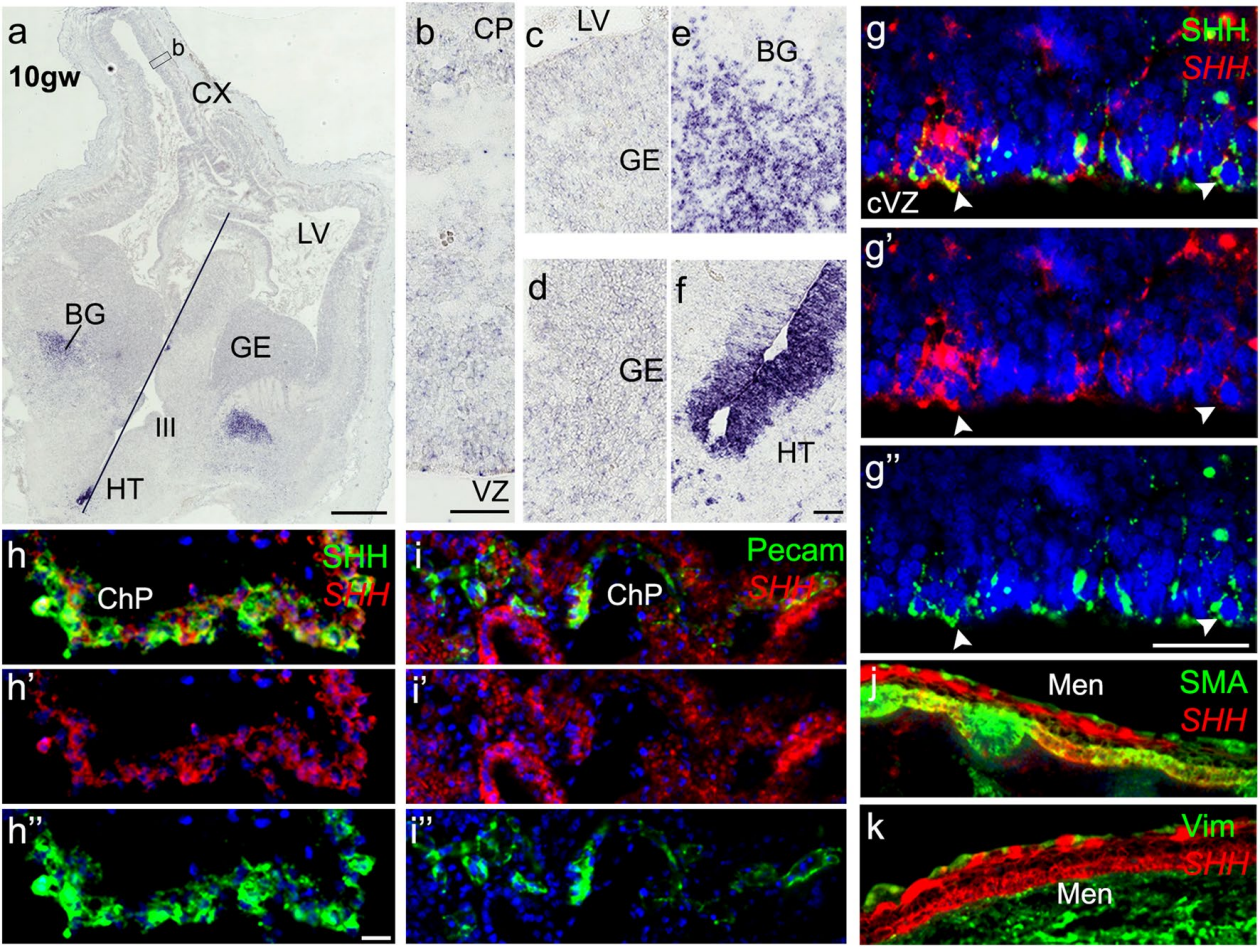
Expression of Sonic Hedgehog (*SHH*) in the human brain at 10 gw. **a** Coronal sections through both cerebral hemispheres show *SHH* mRNA signal in the diencephalon (hypothalamus, HT) and basal ganglia (BG). The line indicates the midline. **b** Low-level *SHH* expression in the developing cortex, mainly in the ventricular zone (VZ) and cortical plate (CP). **c, d** Scattered cells weakly expressing *Shh* in the VZ of the ganglionic eminence (GE) (**c**) and in other regions of the GE (**d**). **e, f** High-level expression in the future basal ganglia (**e**) and hypothalamic midline (**f**). **g–g″** Both SHH protein (green) and transcript (red) are demonstrated in the cortical VZ in radial glial cells (arrowheads). **h** Choroid plexus expresses *SHH* mRNA (red) and protein (green); single channels (**h–h″**). **i** Pecam (CD31) immunostaining shows that endothelial cells in the choroid plexus  do not express  *SHH* mRNA. **j** SHH-expressing cells stain with antibody to smooth-muscle actin (green) but not vimentin (green) (**k**). Scale bars: **a** 1 mm, **b, f** and **g** 50 µm, **h** 20 µm

**Fig. 2:**
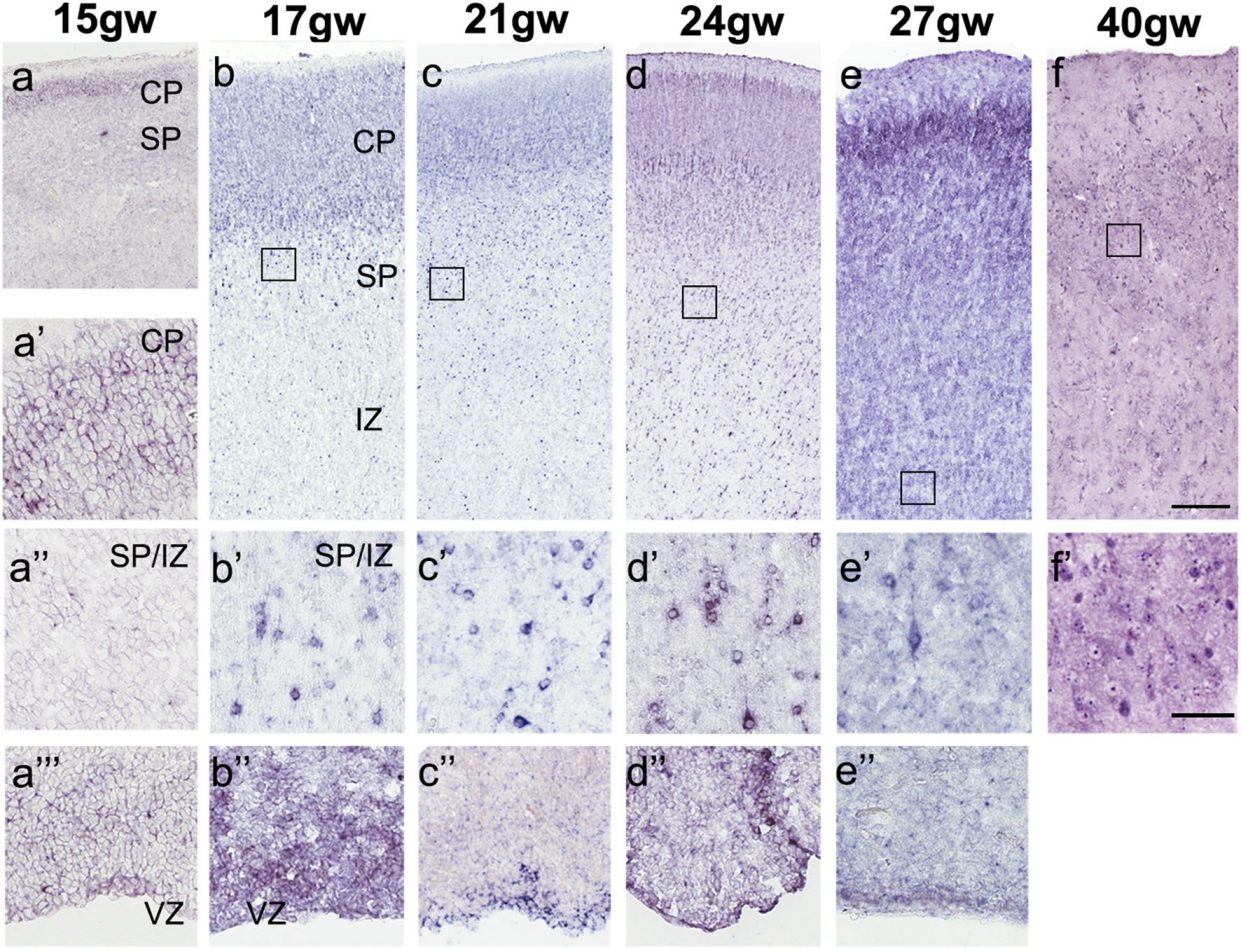
Spatiotemporal changes in SHH expression in the human cerebral cortex between 15 and 40 (newborn) gw. **a–f** Low magnification of the cortical column at the indicated age. **a″–f′** High magnification of cells in the subplate (SP)/intermediate zone (IZ) area (indicated boxes). **a‴–e″** High magnification of cells in the cortical VZ (except at 40 gw,when there is no VZ). *CP* cortical plate. Scale bars : **f** 200 µm, **f′** 50 µm

Detailed information on the spatial distribution of SHH in the human developing brain is lacking, in part due to the limited availability of tissue and the failure of most commercially available antibodies to label SHH. We thus used ISH against the human coding sequence of *SHH* to follow expression during 7 months of the human gestational period (8–40 gw; Table S1). The expression of *SHH* was highly dynamic, in accordance with the transcriptomics data available from the Allen Brain Institute (Fig. S1a, b). *SHH* expression in the fetal forebrain increased over the course of development and shifted from the proliferative ventricular (VZ) and subventricular zones (SVZ) (Fig. 1) to the overlying cortical layers (Fig. 2). At the earliest age studied (8–10 gw), Shh mRNA was predominantly restricted to the ventral forebrain, including the midline of the hypothalamus and thalamus, and to the emerging basal ganglia (Fig. 1a, e, f), in a similar pattern detected in the early embryonic mouse brain (Fig. S6a). By contrast, *SHH* mRNA was only weakly expressed dorsally, in the cortical plate (CP) and cortical VZ (Fig. 1a, b), a result confirmed by TSA-FISH (tyramide signal amplification) and the absence of a signal with the sense probe (Fig. 1g′ and S5). Furthermore, immunostaining with the rabbit monoclonal anti-SHH antibody (Farmer et al. 2016) revealed the definite presence of SHH protein in this area (Fig. 1g, g′′ and Fig. S1c–c′′). The proximal choroid plexus (ChP) was also positive for the *SHH* transcript and protein (Fig. 1h–h′′). Notably, the levels of SHH were much higher in ChP cells than in the nearby VZ, as observed by the differences in signal intensity at the different exposure times/settings (Fig. S1c–c′′). Thus, it cannot be ruled out that the positive signal for the SHH protein in the cortical VZ reflected the uptake of ChP-secreted SHH by RGCs. However, SHH immunostaining in the cortical VZ appeared in more frontal sections that are much further from the ChP (not present in the same section), suggesting that SHH is produced locally.

Another source of SHH protein in the developing human brain at this stage is the meninges, which were strongly positive for *SHH* mRNA and protein. Within the meninges, *SHH*-expressing cells were those of smooth-muscle lineage, rather than fibroblasts, as demonstrated by co-localization with smooth-muscle actin but not vimentin antibody (Fig. 1j, k). Finally, SHH mRNA was detected in the endothelial cells of blood vessels in the GE stained for the endothelial cell marker PECAM (CD31), indicating that the developing vasculature is an additional source of SHH (Fig. S1d–d′′). Notably, endothelial cells in the ChP did not express SHH (Fig. 1i). Extracortically, *SHH* mRNA co-localized with SHH protein in the hypothalamic midline and retinal ganglion cells. In addition to validating the specificity of the antibody, this result provided insight into the range of SHH diffusion from its source (Fig. S1e–e′′, f–f′′).

In the subsequent stages of development (15–17 gw), the density and distribution of *SHH* transcripts increased considerably throughout the forebrain (Fig. 2a, b; Fig. S2a). Strong signal was detected not only in the cortical VZ/SVZ (Fig. 2a′′′, b′′; Fig. S2a), but also in the cortical plate (CP) (Fig. 2a′, b; Fig. S2a), whereas the signal in the intermediate zone (IZ) and subplate (SP) increased dramatically from 15 to 17 gw (Fig. 2a′, b′).

**Fig. 3:**
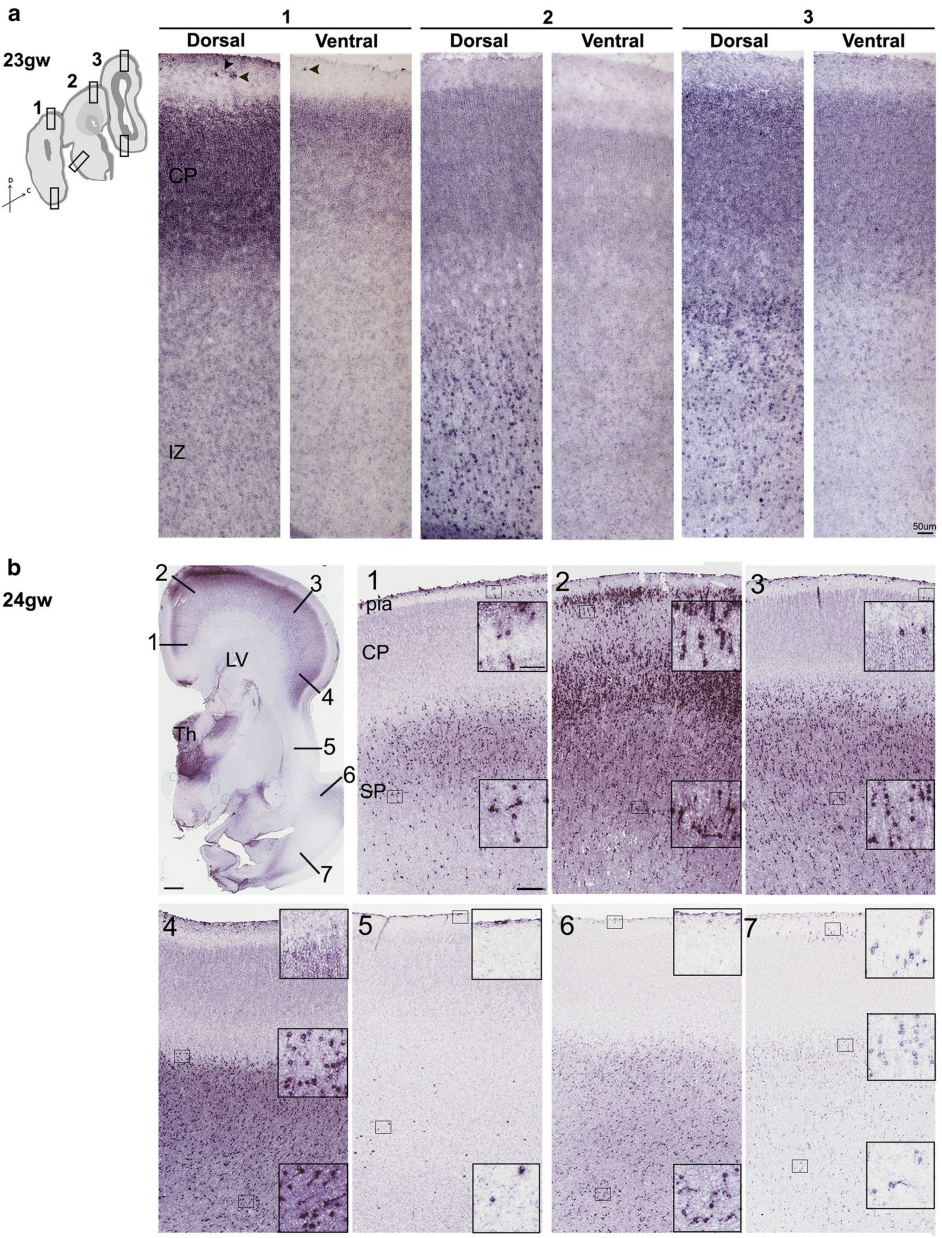
Gradients of *SHH* mRNA in 23–24 gw brains. **a** Coronal sections from three tissue blocks (1–3) from frontal to occipital pole show rostro-caudal gradient of *SHH* mRNA expression. In addition, dorsal areas show higher expression than ventral in each block. **b** In a single coronal section, the expression of *SHH* transcripts shows pronounced differences (1–7). Boxed ares show higher magnification along the cerebral cortex. 1 Cingulum; 2–3 dorsal cortex, future somatosensory or motor areas; 4 frontal, preinsular cortex; 5 insula; 6–7 temporal cortex. Scale bars: **a** 50 µm, **b** 2 mm, (1) 250 µm and inset 50 µm

In the next stage of development, at mid-gestation (18–24 gw), *SHH* expression increased steadily in subpopulations of cells in the expanded outer SVZ (oSVZ) (Figs. 3a, 6f), and in the IZ and SP, the zones through which neurons migrate (Figs. 2, 3a). At the same time, a very strong signal was present in cells located in the ganglionic eminence (GE) and hippocampus (Fig. 6j, S7c and Fig. S3). From 24 gw onwards, the *SHH* transcript signal in the cVZ decreased progressively, coinciding with a reduction in the size of these structures in late fetal development. This is the first study demonstrating SHH expression in the GE and cVZ| in stages after 14 gw. *SHH* expression in the upper cortical layers remained high between 24 and 27 gw and persisted in the newborn (40 gw), in which SHH-expressing cells were found in all layers of the cerebral cortex (Fig. 2) and in the hippocampus (Fig. S3). A similar expression pattern was detected in mouse postnatal day 4 (P4) brain (Fig.S7d–d′′).

We then examined whether *SHH* transcripts were present uniformly along the rostro-caudal and dorso-ventral axis or formed a gradient in these regions of the human fetal cortex. ISH performed in sagittal sections of 15-gw forebrain showed that, although *SHH* expression appeared slightly higher in the rostral and caudal cortical areas than in the dorsal and ventral areas, the difference over the entire cortex was not significant (Fig. S2).

However, in later developmental stages (21–24 gw), *SHH* expression exhibited a high rostral to lower caudal gradient, as illustrated in coronal sections prepared from rostral (frontal), medial, and caudal (occipital) tissue blocks of the 23-gw fetal forebrain (Fig. 3a). Moreover, within each tissue block, *SHH* signal intensity was consistently stronger in dorsal than in ventral cortical areas (Fig. 3a).

In addition, cortical *SHH* expression exhibited regional differences, as illustrated in single coronal sections prepared through the level of the thalamus. The signal was always stronger above the insular region of the cortex, in the prospective somatosensory/motor cortices and possibly including Broca’s area (Fig. 3b:1–4), than in the ventrally positioned temporal cortex (Fig. 3b:6–7). This pattern, which first emerged around 19 gw and became prominent at 22–24 gw, confirms the transcriptome data (Fig. S1a, b) that suggest higher expression in the dorsal than ventral cortex for this developmental stage.

These results revealed the complex pattern of *SHH* expression in the developing cortex and thus a possible role for *SHH* in cortical arealization during the second and third trimesters of gestation.

### Characterization of SHH-expressing cells during mid-gestation

**Fig. 4:**
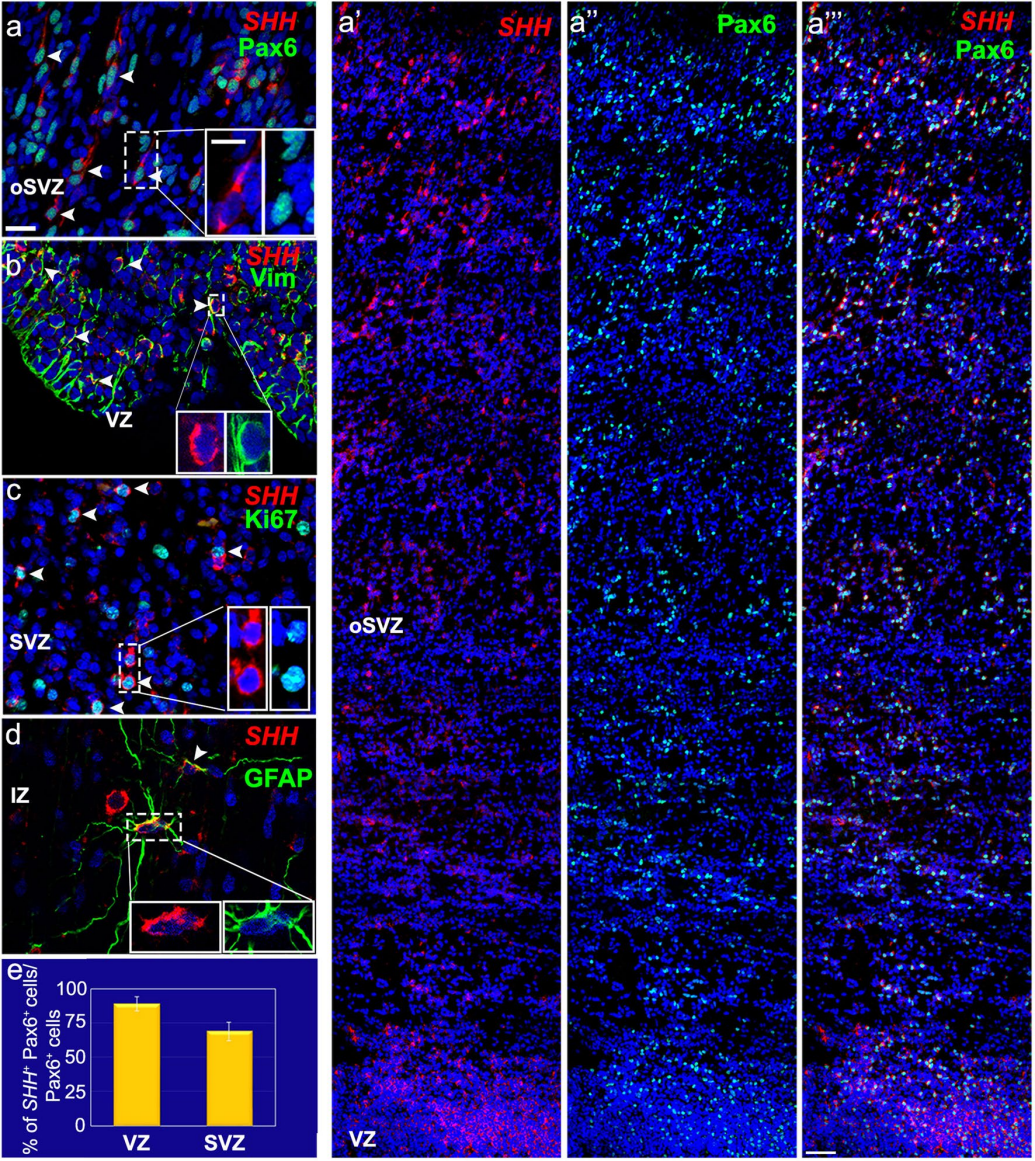
Cell-type-specific expression of *SHH* transcript in the 18–24-gw fetal brain. **a′–a‴** Combined ISH for *SHH* (red) and immunostaining for Pax6 (green) show numerous radial glial cells (RGCs) that co-localize *SHH* transcript and Pax6 in the proliferative zones (VZ and subventricular zone, SVZ). **a**-inset Higher magnification of co-labeled cells in the outer SVZ (oSVZ) (arrowheads). **b** Vimentin staining for RGCs (green) after ISH for *SHH* (red) confirms that RGCs in the VZ express *SHH* (arrowheads). **c** Numerous proliferating *SHH* ^+^ cells, indicated by Ki67 co-expression. The inset shows a higher magnification of the boxed area. **d** Mature astrocytes (GFAP^+^) in the IZ/SP express *SHH* mRNA. **e** Percentage of total Pax6^+^ cells that co-express *SHH* (red) in the VZ (89% ±5.19 SEM) and SVZ (69% ± 6.6 SEM) of 22–24-gw tissues (*n* = 3). Scale bars: **a** 25 µm, **a**-inset 10 µm, **a‴** 50 µm

After establishing that *SHH* mRNA is expressed widely in fetal brain, we asked which cell types express *SHH* in the developing human cortex. The answer to this question is important, because it might indicate the roles played by SHH during development. We, therefore, subjected the fetal tissue sections to FISH followed by immunohistochemistry with cell-type-specific markers. Specifically, we asked whether during the late second trimester of gestation (20–24 gw). *SHH* transcripts are expressed by cortical progenitors, that is, RGCs, which are the predominant cell type in the proliferative VZ/SVZ. Using the RGCs markers Pax6, GFAP, and vimentin, we identified cells that co-express *SHH* mRNA as well as these markers (Fig. 4). Moreover, many of the *SHH*-expressing cells were proliferating, based on their co-labeling for the proliferation marker Ki67 (Fig. 4c). Quantification of the double-labeled cells showed that 89% (± 5.19 SEM, *n* = 3) of the Pax6-positive cells in the VZ expressed *SHH* transcripts, compared to 69% (± 6.6 SEM, *n* = 3) in the SVZ; thus, at this developmental stage the majority of RGCs in the proliferative zones produced SHH (Fig. 4e).

**Fig. 5:**
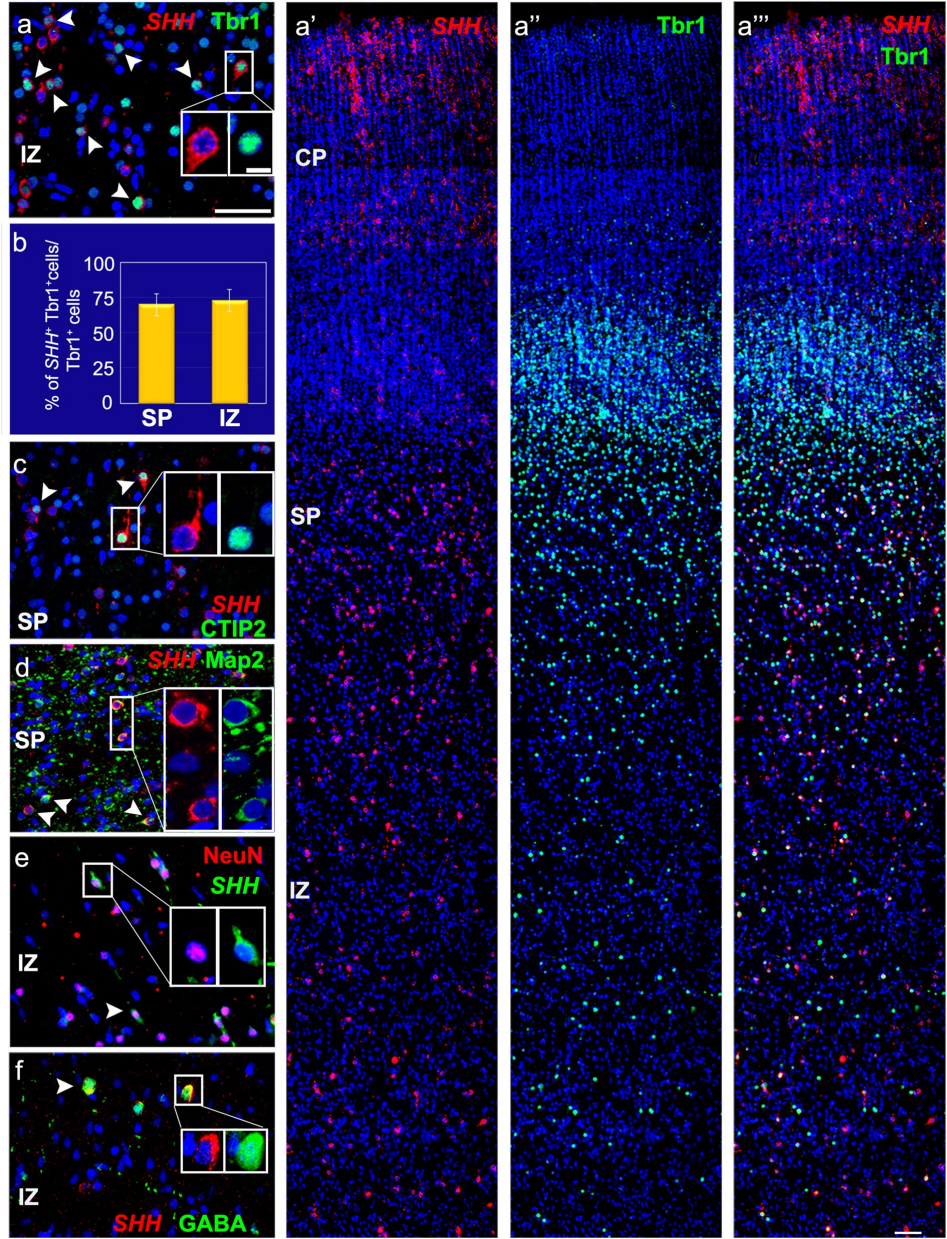
Various subtypes of neurons express *SHH* in the 18–24 gw cortex. **a–a‴** Co-labeling for *SHH* mRNA (red) and Tbr1 (green) reveals many postmitotic projection neurons across the SP and IZ expressing *SHH*. **a′, a″** Single channels. **b** Percentage of Tbr1^+^ cells expressing *SHH* in the SP (70% ±7.6 SEM) and IZ (73% ±7.8 SEM) in 22–24-gw sections (*n* = 3). **c–f** *SHH* is expressed by neurons labeled with CTIP2 (**c**), MAP2 (**d**), NeuN (**e**), and GABA (**f**). Scale bars: **a** and **a‴** 50 µm, **a**-inset 10 µm

Since progenitors in the fetal cortex give rise mainly to glutamatergic neurons, SHH-expressing cells in cortical layers were probed with the glutamatergic cell marker Tbr1 (T-box Brain 1) to determine whether they belong to this neuronal population (Fig. 5a–a′′′, b). From the total population of Tbr1^+^ cells in the subplate and the IZ, around 70% (*n* = 3) were positive for *SHH* (Fig. 5b). Thus, in addition to RGCs in the VZ/SVZ, a subpopulation of glutamatergic neurons in cortical layers is source of SHH. This finding was confirmed using another marker for deep cortical layer neurons, CTIP2 (Fig. 5c). Two more general neuronal markers, Map2 and NeuN, were also co-expressed with *SHH* transcripts in a subpopulation of cortical neurons (Fig. 5d, e).

Co-labeling with GABA (Fig. 5f) or Gad65/67 (Fig. S4a, a′) suggested that at mid-gestation, cortical interneurons are less likely to express *SHH* transcript than glutamatergic neurons. Since not all *SHH* mRNA^+^ cells overlap with neuronal markers in higher cortical layers, we investigated whether non-neuronal populations in the cortex also express *SHH* transcript. Indeed, GFAP^+^ astrocytes in the IZ and SP/CP expressed *SHH* (Fig. 4d), but not oligodendrocytes or microglia labeled with Olig2 or Iba1, respectively (Fig. S4b, c). These data are consistent with a heterogeneous cell population producing Shh during mid-gestation, with the two main sources being RGCs and glutamatergic neurons.

### SHH-signaling pathway in the developing human cortex

**Fig. 6:**
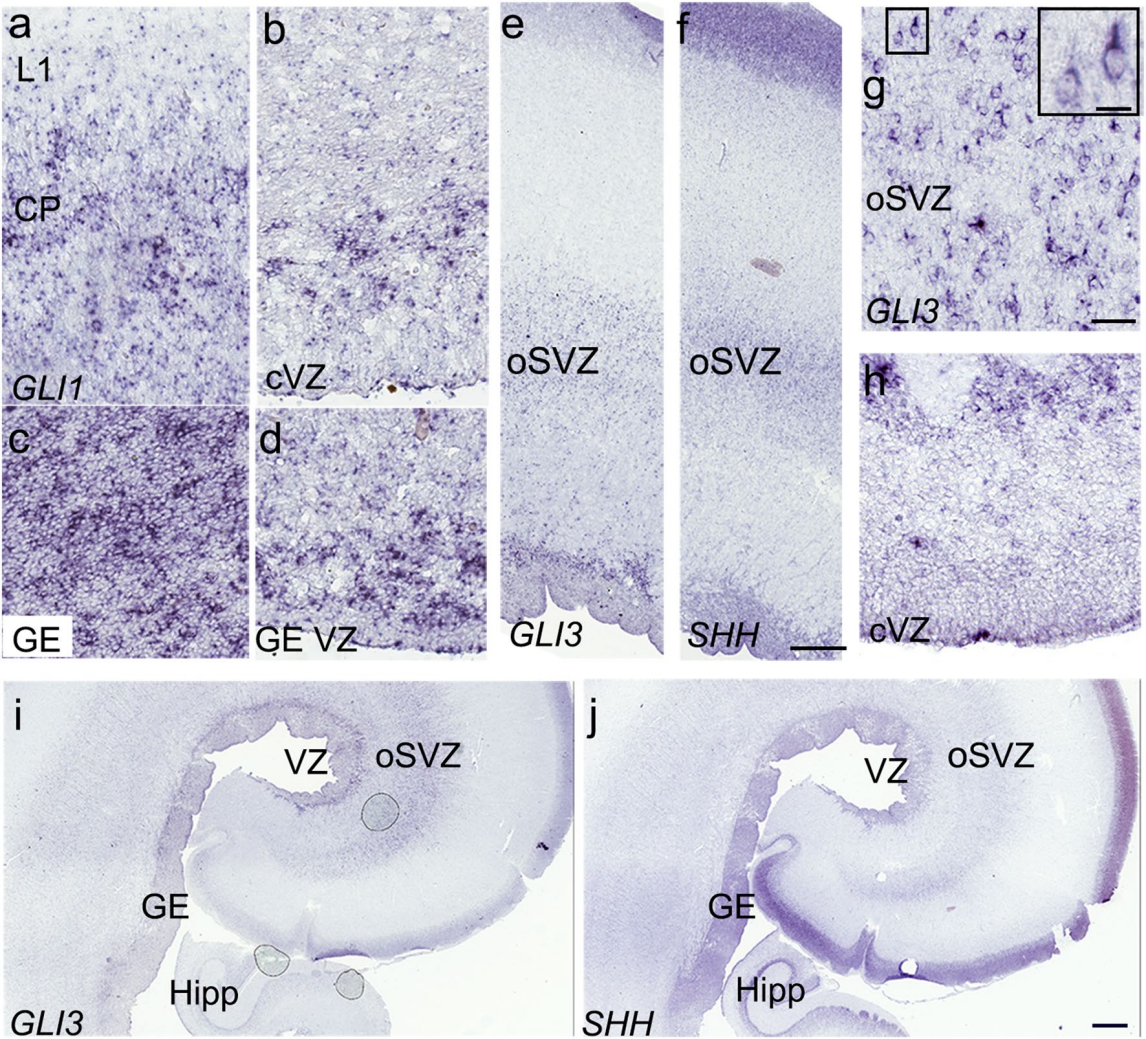
Components of the SHH-signaling pathway are expressed in the human fetal cortex at 22 gw. **a, b** ISH reveals the cortical expression of *GLI1* (CP and VZ), indicative of SHH-signaling activity in these areas. **c, d** Strong *GLI1* expression in the GE suggests higher levels of SHH activity ventrally. **e, f** *GLI3* and *SHH* transcripts show similar high-density signals in the VZ and oSVZ. **g** On higher magnification, GLI3^+^cells in the oSVZ resemble RGCs and are more numerous than in the VZ (**h**). **i, j** Comparison of the *GLI3* and *SHH* expression patterns in adjacent coronal sections at the level of the hippocampus (Hipp) of the 22 gw fetal brain. Scale bars: **f** 500 µm, **g** 50 µm, **g**- inset 15 µm, **j** 1 mm

Despite the demonstration of the widespread expression of *SHH* in the developing human cortex, whether the classical *SHH* pathway was activated remained to be tested. Gli1 (GLI zinc finger transcription factor), a transcription factor activated by Shh-signaling, is the most sensitive and reliable read-out for this pathway as, unlike Gli2 and Gli3, it acts only as an activator (Bai et al. 2004). The presence of *GLI1* would, therefore, provide proof that the expressed SHH activates the signaling pathway in nearby cells. ISH using the human *GLI1* probes on sections from fetuses age 19–24 gw found that the *GLI1* transcript was expressed in the cortical VZ and CP/SP as well as in the GE (Fig. 6a–d). The intensity of the GE signal was much stronger, indicating a higher level of *SHH* expression, and hence SHH-signaling activity, in this area. *GLI3*, a transcription factor that acts as both a repressor and an activator of the Shh pathway (Ingham and McMahon 2001), was highly expressed in the cortical VZ, in the inner and outer SVZ (Fig. 6e, g–h), and in the hippocampus (Fig. 6i), confirming previously published transcriptomics data showing that *GLI3* is expressed by human RGCs (Pollen et al. 2015).

**Fig. 7:**
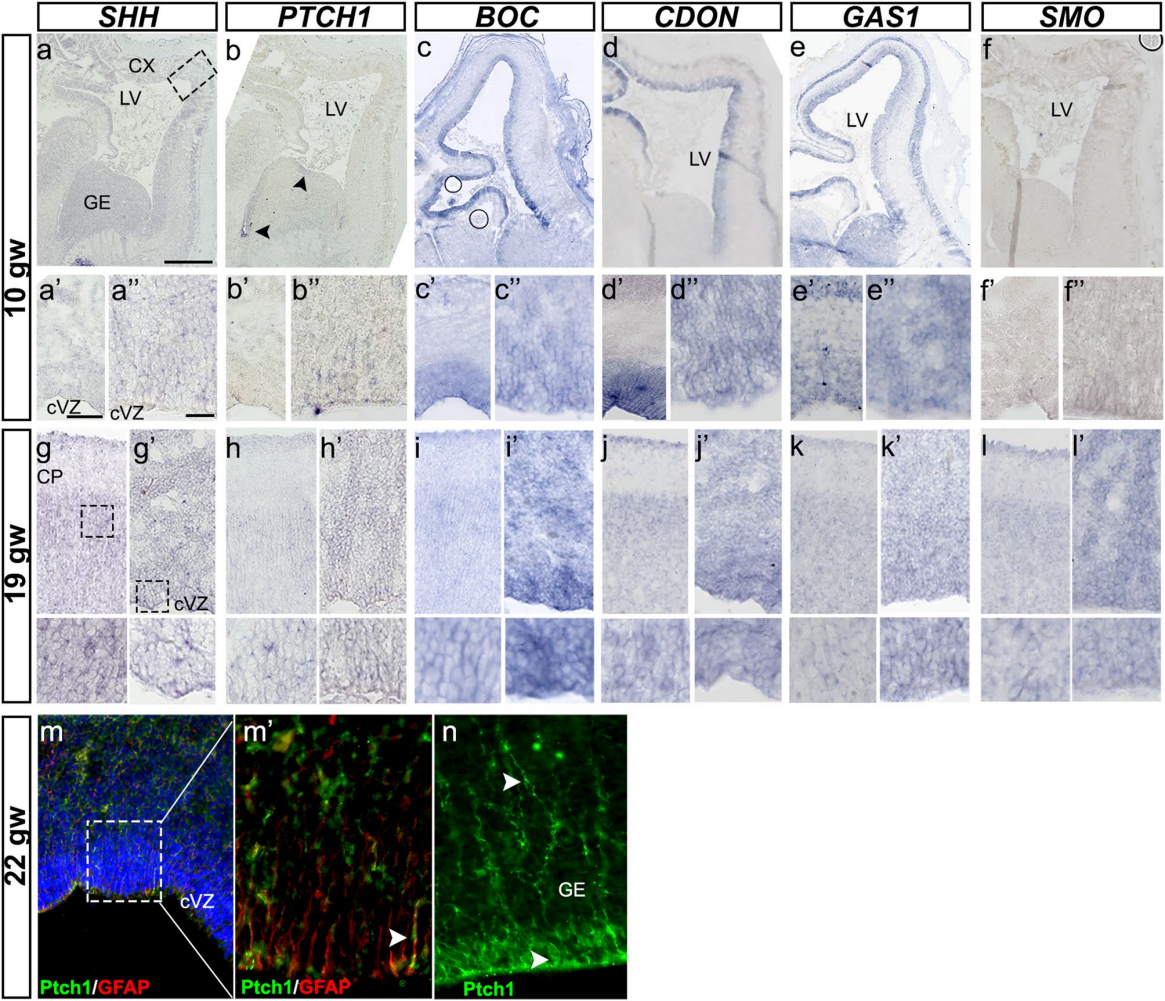
Expression of SHH receptors in the fetal human brain at 10 and 19 gw. **a–a″** ISH for *SHH* on a coronal section from a 10-gw brain, as a reference for all SHH receptors in sections from the same fetus. Higher magnification of the boxed areas in (**a**): **a′** cortex, **a″** cVZ. Weak signal is detected in the cortical VZ and GE. **b–b″** ISH for *PTCH1* receptor reveals very low expression in the VZ (**b″**). *BOC* expression is much more prominent along the cortical VZ and GE VZ (**c–c″**), whereas *CDON* expression is restricted to the cortical VZ (**d–d″**). *GAS1* is the only receptor expressed in both the VZ and CP, in addition to the GE (**e–e″**). *SMOOTHENED* (*SMO*) is hardly detected in the VZ at this stage (**f–f″**). **g–l′** *SHH* and all its receptors in contiguous sections of 19-gw brain. Two areas are presented for each gene: CP and VZ/SVZ. **m** Co-staining of 22-gw brain with Patched1 and GFAP antibodies shows that RGCs in the cortical VZ express this receptor. **m′** Higher magnification of the boxed area in (**m**). **n** Ptch1 immunoreaction in the same section reveals a strong expression in the GE. **a** 1 mm, **a′** 150 µm, **a″** 25 µm

We then asked whether the SHH receptor PATCHED1 (PTCH1) is expressed in the vicinity of *SHH*-expressing cells. We tested tissues from fetuses of two gestational ages, early (10 gw), when cell proliferation, migration, and neurogenesis/fate specification are predominant, and later, at mid-gestation (19–24 gw), when additional processes such as axonal guidance and synaptogenesis occur. At 10 gw, *PTCH1* transcripts were non-detectable in the neocortex (Fig. 7b, b′), while expression was low in the VZ of the GE and in the thalamic and hypothalamic neuroepithelium, paralleling the spatiotemporal pattern of strong *SHH* expression in these ventral regions. At later stages (19–24 gw), when *SHH* expression increased in the cortex, *PTCH1* was seen in the cortical VZ, although at much lower levels than in ventral areas such as the GE or hypothalamus (Fig. 7h, h′). Immunostaining with PTCH1 antibody verified low- and high-level expression in the cortical VZ and GE, respectively (Fig. 7m, n). Co-labeling with GFAP also demonstrated the expression of this receptor by RGCs in the cortical VZ (Fig. 7m′). A weak *PTCH1* mRNA signal was detected in the dentate gyrus of the hippocampus (Fig. S3h).

In addition to Ptch1, three other membrane-associated proteins, the structurally related Boc (biregional Cdon-binding) and Cdon (cell-adhesion-molecule-related/down-regulated by oncogenes) proteins and the vertebrate-only Gas1 (growth-arrest-specific 1) protein, are thought to function as positive modulators of Shh signaling by enhancing the presentation of Shh to its receptor, Ptch1 (Tenzen et al. 2006; Allen et al. 2007; Martinelli and Fan 2007). Both Boc and Cdon promote Shh-dependent cell-fate specification and axon guidance (Tenzen et al. 2006; Okada et al. 2006), while Gas1 regulates the ventral specification of neural tube progenitors (Allen et al. 2007; Martinelli and Fan 2007) and CGNP (cerebellar granule neural progenitors) proliferation (Liu et al. 2001). Boc, Gas1, and Cdon are required for successful Shh signaling (Izzi et al. 2011). We found that *BOC* and *GAS1* were strongly expressed at 10 gw in cortical VZ and GE, whereas *CDON* was present only in the cortical VZ (Fig. 7c–e). *GAS1* was also expressed in the CP at this stage. The ChP cells, however, express only *BOC* at 10 gw (Fig. S8b). At later stages (19–24 gw), the expression of *BOC* and *CDON* was similar to that in earlier stages: high in the cortical VZ and SVZ and low in the CP/SP and GE (Fig. 7i–i′, j–j′). *GAS1* expression in the CP was lower than at 10 gw, but remained high in the cortical VZ, oSVZ, and GE (Fig. 7k–k′). Finally, expression of the SHH signal transducer, *SMO* (smoothened) was weak in the 10-gw brain (Fig. 7f, f′′), but increased in the 19-gw cortical VZ and in the CP (Fig. 7l–l′). *SMO* expression was also detected in the 10-gw ChP (Fig. S8a). Thus, in summary, all three receptors (*BOC, CDON*, and *GAS1*) were expressed in the VZ during early fetal development, pointing to their role in cell proliferation. *CDON* and *GAS1* were expressed at higher levels than *BOC* in the CP, suggesting their additional roles in cortical development.

## Discussion

SHH is crucial for human brain development and changes in its signaling lead to distinct neuropathologies (Heussler et al. 2002; Nanni et al. 1999; Belloni et al. 1996; Odent et al. 1999; Santiago et al. 2006; Currier et al. 2012). Despite its importance, information on *SHH* expression profile in the developing human brain is still fragmentary. Here, we provided evidence of the expression of *SHH* and the transcription factors, and receptors necessary for its signaling, in specific cortical layers and cell types. Our study spans the course of most of the gestational period. The spatiotemporal distribution suggests the involvement of SHH in diverse developmental processes in the human telencephalon, including cell proliferation and cell-fate specification in the VZ/SVZ, the subsequent migration of newly generated neurons, synaptogenesis, and circuit formation.

Our results agree with those of the previous studies (Odent et al. 1999), in which *SHH* mRNA was mostly detected in the ventral structures of the human brain during early stages (8–10 gw), with very low signal in the developing cerebral cortex. Given that a high concentration of SHH is known to induce patterning and cell-fate specification, whereas low levels regulate proliferation (Komada 2012; Komada et al. 2008; Wang et al. 2016a, b) this pattern of signal localization suggests that SHH regulates patterning and cell-fate specification in the human ventral forebrain and proliferation in the dorsal forebrain. *SHH* transcripts were clearly detected in the cortical VZ/SVZ at 15 gw, which is consistent with our previous in vitro results showing that this morphogen is secreted by dorsal RGCs and induces their proliferation (Radonjic et al. 2016). The function of Shh in proliferation and regulation of symmetric/asymmetric divisions of intermediate progenitors in mice (Dave et al. 2011) points to a role as a mitogen in the human cortical proliferative zone. This role is probably maintained even past mid-gestation, since neurogenesis in humans continues up to 27 gw, later than previously known (Malik et al. 2013). Between 15 and 27 gw, *SHH* was also expressed in the IZ, SP, and CP, where the intensity of the signal increased over time. Furthermore, we observed robust inter-regional and areal differences in the expression of *SHH* during mid-to-late gestation in fetal neocortex. Although it is difficult to interpret this spatiotemporal expression pattern, these differences most likely have biological origin and they are not due to tissue quality or other variation as observed in all cases studied. The *SHH*-positive cells in developing cortical layers were mostly neurons expressing Map2 or NeuN, with a subpopulation expressing glutamatergic markers (Tbr1 or CTIP2), as previously reported for the early postnatal mouse brain (Harwell et al. 2012). The SHH transcript was also identified in a subpopulation of GABAergic cells, in accordance with the results of a single study in mouse (Komada et al. 2008). Given that Shh in mice plays a role in neuronal migration, axonal guidance (Fuccillo et al. 2006; Baudoin et al. 2012; Bourikas et al. 2005; Charron et al. 2003; Yam et al. 2009), and synaptic connectivity (Harwell et al. 2012), it is likely that it has similar functions in the developing human fetal neocortex. Moreover, the finding that GFAP-labeled astrocytes in the upper IZ, SP, and CP were positive for SHH suggests a role in numerous processes in which astrocytes are involved, such as synapse formation and synaptic plasticity (Farmer et al. 2016; Eroglu and Barres 2010) as well as the formation and maintenance of the blood–brain barrier (BBB). Indeed, recent work demonstrated that Hedgehog signaling promoted the formation and integrity of the BBB as well as the immune quiescence of the central nervous system (Alvarez et al. 2011). Microglia and oligodendrocytes were negative for *SHH*, but Olig2^+^ cells were found in close proximity to S*HH*-expressing cells, which correlates well with our previous in vitro study showing that SHH promotes the generation and maintenance of forebrain Olig2 progenitors (Ortega et al. 2013).

Our finding of *GLI1* in the fetal cortical VZ and CP suggests active SHH signaling in the developing human cortex. This result is in line with our previous demonstration of functional SHH signaling in cortical RGCs in vitro, as indicated by the increased levels of *GLI1* and *PTCH1* after SHH treatment (Radonjic et al. 2016). In the present study, we provided evidence of PTCH1 (protein) as well as *BOC, GAS1*, and *CDON* expression in the human RGCs of the cortical VZ, which suggest an autocrine function for SHH in these progenitors. The role of these receptors has not been studied in cortical development, but they are known as positive modulators of Shh signaling in mice, in both cell proliferation in the cerebellum (Liu et al. 2001; Izzi et al. 2011) and cell-fate specification in neural tube progenitors (Allen et al. 2007; Martinelli and Fan 2007). A recent study has shown that Boc, Cdon, and Gas1 are necessary components of the Shh receptor complex and essential in Shh signal transduction in vertebrates (Izzi et al. 2011). Their strong expression in the human cortical VZ in association with the low-level expression of *SHH* and *PTCH1* during early stages (10 gw) suggests that they could act as enhancers of SHH signaling. Impaired function of *BOC, CDON*, and *GAS1* appears to underlie holoprosencephaly, both in humans (Clement et al. 2007; Ribeiro et al. 2010; Pineda-Alvarez et al. 2012; Bae et al. 2011) and in mice (Seppala et al. 2007; Zhang et al. 2006; McLellan et al. 2008). Interestingly, we found that *BOC* is strongly expressed in the human, but not in the mouse cortical VZ (Fig. S6, brainatlas.org), suggesting a species-specific difference. Indeed, comparative epigenetic profiling of human, monkey, and mouse brain tissue identified epigenetic gains (promoters and enhancers with gained activity) in genes involved in human corticogenesis, including *BOC, SHH, NKX2.1, PTCH1*, and *GLI3* (Reilly et al. 2015).

Recent studies have suggested that the transcriptional programs associated with interneuron development in human are very similar in the GE and cortical VZ (Miller et al. 2014), which points to a role for SHH in interneuron fate specification, in addition to cell proliferation, in the cortical VZ. Indeed, our previous in vitro results show that SHH affects the commitment of some cortical RGCs to interneuronal fate (Radonjic et al. 2016). This points to the need for a better understanding of the origin and development of human cortical interneurons (Radonjic et al. 2014; Alzu’bi et al. 2017; Clowry et al. 2010). Such studies are likely to provide important insights into the pathogenesis of human neuropsychiatric disorders such as schizophrenia, in which dysfunction of GABAergic interneurons has been implicated (Benes and Berretta 2001; Guidotti et al. 2005; Lewis et al. 2005; Selemon and Zecevic 2015).

In conclusion, the present study fills the gap in our knowledge about the presence of SHH in the developing human cortex which may enhance our understanding of human corticogenesis and the pathologies associated with defective SHH signaling.

## Electronic supplementary material

Below is the link to the electronic supplementary material.

Supplementary material 1 (DOCX 15 KB)

Fig. S1 **a** RNA-seq data from http://www.brainspan.org/rnaseq/ show the dynamic expression of Sonic Hedgehog (*SHH*) during brain development in utero. The different cortical areas were grouped in “dorsal” and “ventral” groups to show that during 18–22 gw *SHH* expression is higher in dorsal than in ventral regions. **b** The same data, grouped in the different cortical areas, show the changes in *SHH* expression per area along the gestational timeline. **c–c″** Three different exposure times highlight the difference in *SHH* expression levels between choroid plexus (ChP) cells and the RGCs in the proximal ventricular zone (VZ; arrows). **d–d″** SHH transcript is expressed by blood vessel endothelial cells labelled with the endothelial marker PECAM in the ganglionic eminence (GE) at 10 gw. **e–e″** Co-labeling for SHH mRNA (red) and protein (green) in the hypothalamic midline reveals the specificity of the antibody and the range of diffusion of the secreted protein.**f–f″** Expression of both SHH protein (green) and transcript (red) in the human fetal retina confirms the specificity of the antibody in humans, as previously shown in the mouse (TIF 14429 KB)

Fig. S2 Gradients of SHH mRNA in 15-gw brain. **a** A sagittal section of a 15-gw forebrain shows a slight rostro-caudal gradient, best seen on higher magnification of the boxed areas presented on the right (TIF 7220 KB)

Fig. S3 Expression of SHH in the human fetal hippocampus. **a–c** Distribution of *SHH* transcripts in the hippocampus of 17-, 22-, and 40-gw tissue. **b′–b″″** Higher magnification of the boxed areas illustrated in (b) shows *SHH* expression in the different areas of the 22-gw hippocampus. **d, e** Fluorescence ISH for *SHH* and Pax6 staining reveal that only some of Pax6^+^ cells in dentate gyrus (DG) and CA1 co-express *SHH* in the 22-gw hippocampus. **f** *SHH* is expressed by Tbr1^+^ cells in the DG of the 22-gw hippocampus. **f′** Higher magnification of the double-positive cells in (f). **g–l** Expression of SHH receptors and downstream molecules in the 19-gw hippocampus shown in contiguous sections. Scale bars **a, b** 1mm, **b′** 100µm, **f′** 50µm (TIF 7928 KB)

Fig. S4 **a** Coronal medial section of the 21-gw fetal brain stained for SHH mRNA (blue) and Gad67 protein (brown) reveals co-labeled cells. **a′** Higher magnification of the boxed area in (a). **b** Double-positive cells are not seen in a tissue section from a 23-gw brain treated for SHH (red) followed by Olig2 staining (green). **b′, b″** Higher magnification of the interventricular zone (IZ) and subplate (SP) areas. **c** Microglial cells (Iba1, light blue) and SHH mRNA (red) staining do not co-label cells in the 10-gw human cortex. Scale bars: **a** 150µm, **a′** 100µm, **b′** 50µm (TIF 3754 KB)

Fig. S5 Sense/control in situ for SHH in 10- and 19-gw tissue. Scale bars: 2mm (TIF 9398 KB)

Fig. S6 Expression pattern of Shh-signaling genes in the embryonic mouse brain. Data obtained from the Allen Brain Atlas (TIF 11830 KB)

Fig. S7 In situ hybridizations on mouse brain tissue with the human SHH antisense (AS) and sense (S) probe. Only (b) was probed with the sense probe. Scale bars: **a** 400µm, **c** 500µm, **c’** 100µm, **d** 1mm, **d″** 150µm (TIF 5166 KB)

Fig. S8 Expression of *SMO* and SHH receptors in the 10 gw Choroid Plexus. **a** SMO expression, **b** BOC, **c** GAS1 and **d** CDON. Scale bar: 50µm (TIF 5016 KB)
