# Supplementary material for: Multiple roles of Sonic Hedgehog in the developing human cortex are suggested by its widespread distribution
Source: Brain Struct Funct. 2018 Feb 28;223(5):2361–75. doi: 10.1007/s00429-018-1621-5 (PMC5968052; doi:10.1007/s00429-018-1621-5)
Supplement: Supplementary file 1 — Supplementary material 1 (DOCX 15 KB) [file 429_2018_1621_MOESM1_ESM.docx]

Table S1: Fetal tissue stages (gestational week) used in this study.

| Stage (gestational week) | Number of samples |
| --- | --- |
| 8-10 | 2 |
| 15 | 2 |
| 16 | 1 |
| 17 | 2 |
| 18 | 3 |
| 19 | 2 |
| 20 | 1 |
| 21 | 3 |
| 22 | 3 |
| 23 | 3 |
| 24 | 3 |
| 27 | 2 |
| 40 | 3 |

Table S2. Primers used for cRNA probe synthesis. The sequence and its position in the transcript are indicated.

| Probe name | F Primer 5’-3’(sense) | | R primer 5’-3’(antisense) | | mRNA ID | Position(bp) | |
| --- | --- | --- | --- | --- | --- | --- | --- |
| *SHH* | T3CAGGAAAGTGAGGAAGTCGCT | T7GTAGTCCTCGTCTCCTCGCTG | | NM_000193.3 | | 183-876 |  |
| *GLI1* | T3CCTGAGCCTTATGGAGCGAGG | Sp6TCACTGGAGCTTTAGCACGG | | NM_005269.2 | | 2297-2940 |  |
| *GLI2* | T3CCTGGACACGGCTCATGTGG | T7GTTCTCTTTGAGCAGCGGTGT | | NM_005270.4 | | 1223-1868 |  |
| *GLI3* | T7GCCAGAGCACTTGATGCTCCA | Sp6GCCCACGGTTTGGTCATAG | | NM_000168.5 | | 3767-4439 |  |
| *SMO* | T7AGCAAGATCAACGAGACCATGC | Sp6ACTGGAGTTGCCACAGGGGT | | NM_005631.4 | | 1607-2217 |  |
| *GAS1* | Sp6AGCTCAACCACACGCGCC | T7TCGTCCAGGCCCCCGTCCGA | | NM_002048.2 | | 746-1448 |  |
| *CDON* | Sp6ACGGTGGATTCAAGCCAGTT | T7CCATGAGAGATGCTTCTGCCT | | NM_016952.4 | | 1327-1680 |  |
| *BOC* | T7GATTGCCAGGGACGGCAGTAT | T3ATAGTGTCACAGTGGCTGGA | | NM_033254.3 | | 645-1093 |  |
| *PTCH1* | Sp6AGACCAACGTGGAGGAGCTGT | T7TCCAGGGTCGTGGTGGTGAA | | NM_000264.3 | | 553--1471 |  |

Table S3. Antibodies used in this study.

| Antibody | Company | Species | Dilution |
| --- | --- | --- | --- |
| Pax6 | Millipore | Rabbit | 1:500 |
| Iba1 | Wako | Rabbit | 1:350 |
| CD31/PECAM | Proteintech | Rabbit | 1:200 |
| GFAP | DAKO | Rabbit | 1:1000 |
| Ki67 | DAKO | Mouse | 1:50 |
| Tbr1 | Proteintech | Rabbit | 1:200 |
| CTIP2 | Abcam | Rat | 1:500 |
| GABA | Sigma | Rabbit | 1:1000 |
| Vimentin | Abcam | Rabbit | 1:100 |
| SMA | Sigma | Mouse | 1:500 |
| Olig2 | Chemicon | Rabbit | 1:500 |
| Shh | Genetech | Rabbit | 1:500 |
| NeuN | Chemicon | Mouse | 1:100 |
| Gad65/67 | Millipore | Rabbit | 1:250 |
