# Supplementary figures and images for: Multiple roles of Sonic Hedgehog in the developing human cortex are suggested by its widespread distribution
Source: Brain Struct Funct. 2018 Feb 28;223(5):2361–75. doi: 10.1007/s00429-018-1621-5 (PMC5968052; doi:10.1007/s00429-018-1621-5)

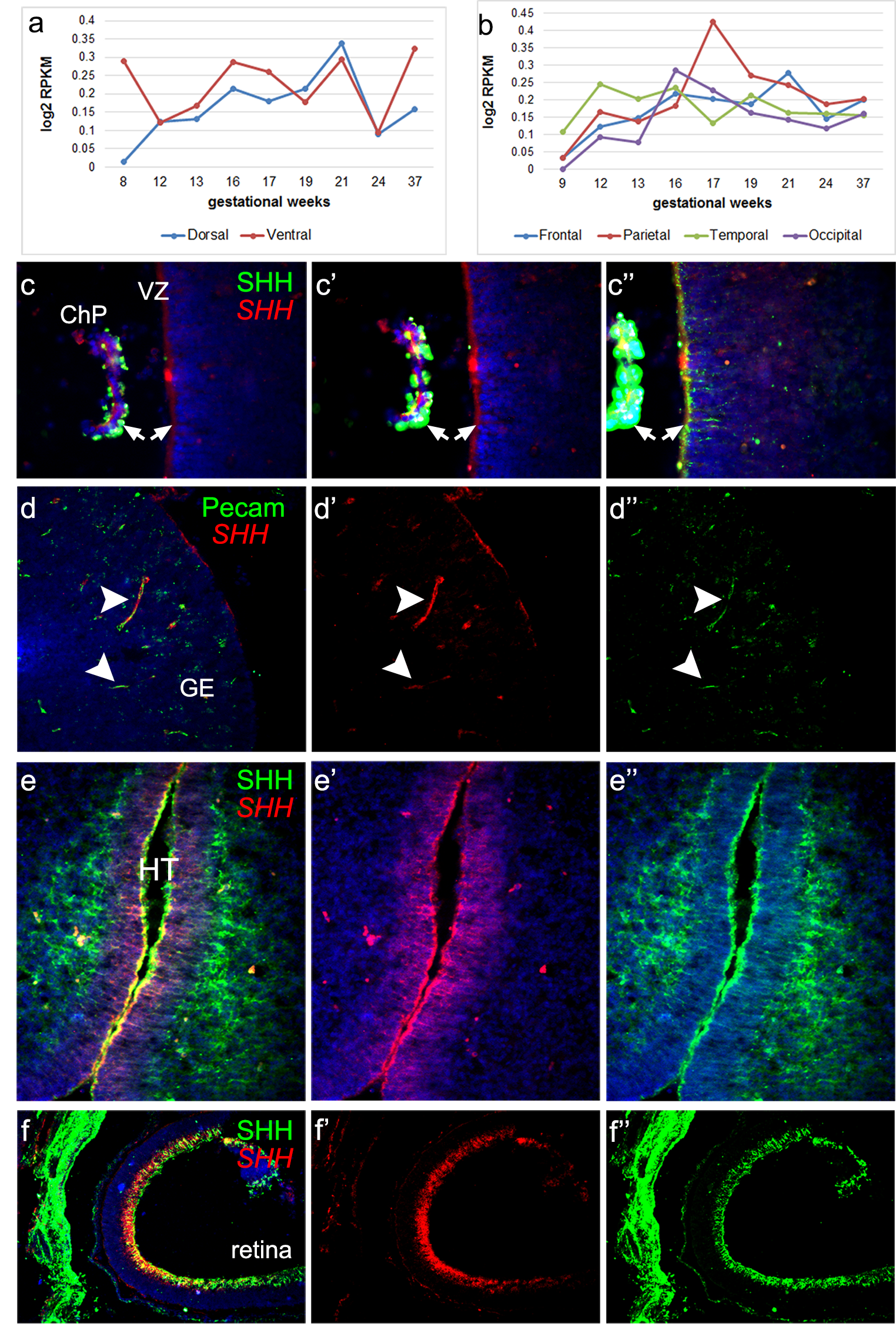

Supplement: Supplementary file 2 — Fig. S1 a RNA-seq data from http://www.brainspan.org/rnaseq/ show the dynamic expression of Sonic Hedgehog (SHH) during brain development in utero. The different cortical areas were grouped in “dorsal” and “ventral” groups to show that during 18–22 gw SHH expression is higher in dorsal than in ventral regions. b The same data, grouped in the different cortical areas, show the changes in SHH expression per area along the gestational timeline. c–c″ Three different exposure times highlight the difference in SHH expression levels between choroid plexus (ChP) cells and the RGCs in the proximal ventricular zone (VZ; arrows). d–d″ SHH transcript is expressed by blood vessel endothelial cells labelled with the endothelial marker PECAM in the ganglionic eminence (GE) at 10 gw. e–e″ Co-labeling for SHH mRNA (red) and protein (green) in the hypothalamic midline reveals the specificity of the antibody and the range of diffusion of the secreted protein.f–f″ Expression of both SHH protein (green) and transcript (red) in the human fetal retina confirms the specificity of the antibody in humans, as previously shown in the mouse (TIF 14429 KB) [file 429_2018_1621_MOESM2_ESM.tif]

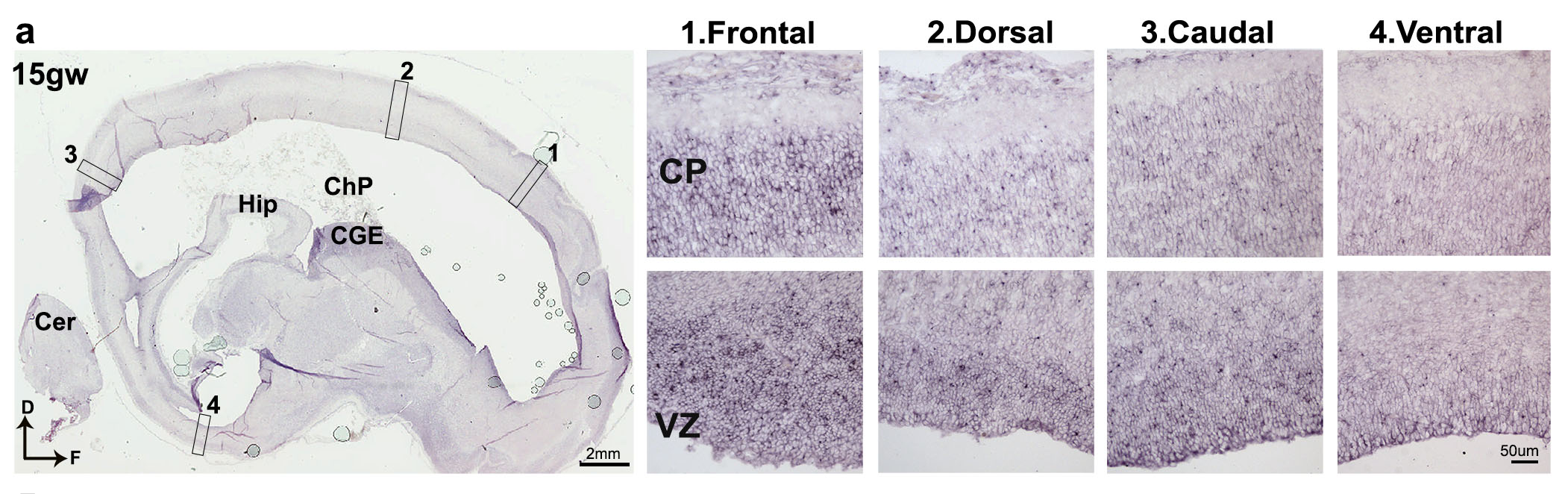

Supplement: Supplementary file 3 — Fig. S2 Gradients of SHH mRNA in 15-gw brain. a A sagittal section of a 15-gw forebrain shows a slight rostro-caudal gradient, best seen on higher magnification of the boxed areas presented on the right (TIF 7220 KB) [file 429_2018_1621_MOESM3_ESM.tif]

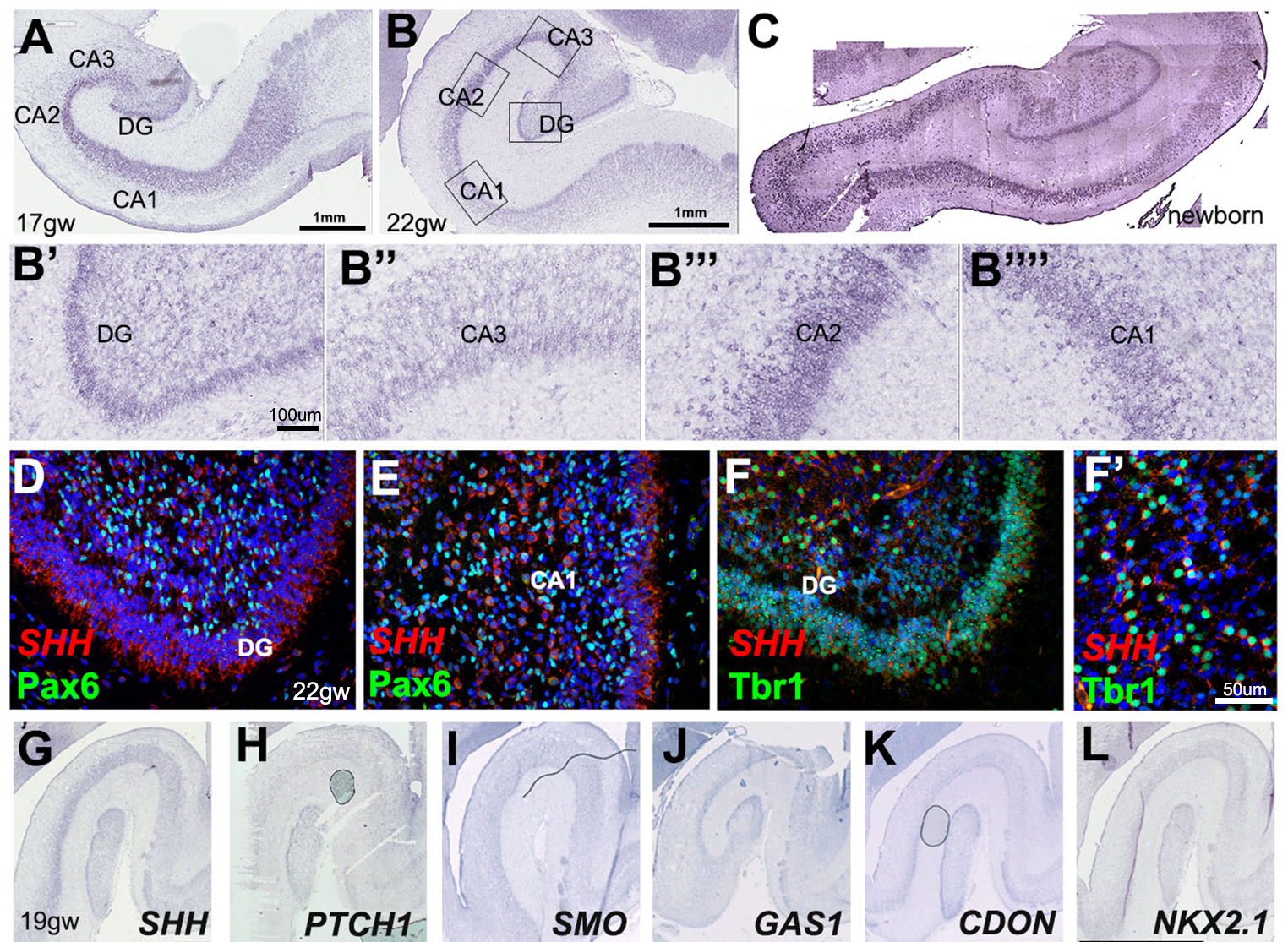

Supplement: Supplementary file 4 — Fig. S3 Expression of SHH in the human fetal hippocampus. a–c Distribution of SHH transcripts in the hippocampus of 17-, 22-, and 40-gw tissue. b′–b″″ Higher magnification of the boxed areas illustrated in (b) shows SHH expression in the different areas of the 22-gw hippocampus. d, e Fluorescence ISH for SHH and Pax6 staining reveal that only some of Pax6+ cells in dentate gyrus (DG) and CA1 co-express SHH in the 22-gw hippocampus. f SHH is expressed by Tbr1+ cells in the DG of the 22-gw hippocampus. f′ Higher magnification of the double-positive cells in (f). g–l Expression of SHH receptors and downstream molecules in the 19-gw hippocampus shown in contiguous sections. Scale bars a, b 1mm, b′ 100µm, f′ 50µm (TIF 7928 KB) [file 429_2018_1621_MOESM4_ESM.tif]

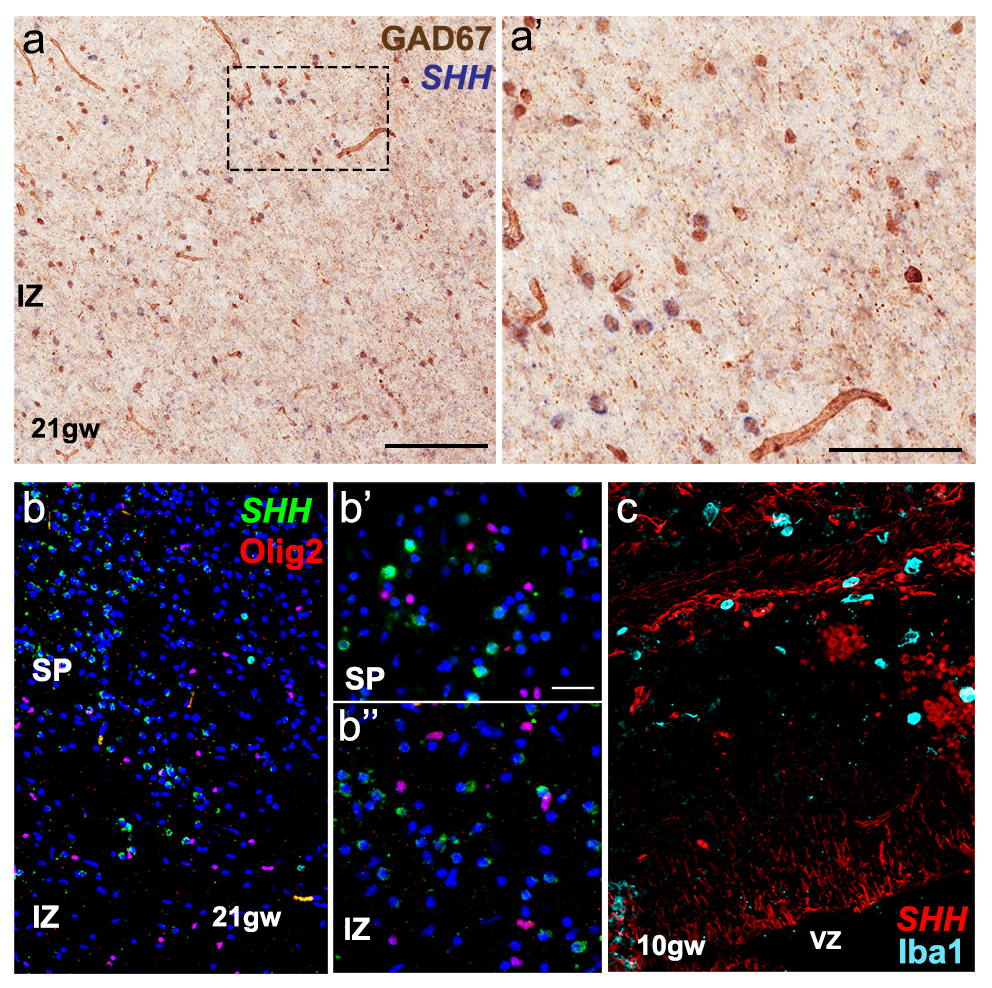

Supplement: Supplementary file 5 — Fig. S4 a Coronal medial section of the 21-gw fetal brain stained for SHH mRNA (blue) and Gad67 protein (brown) reveals co-labeled cells. a′ Higher magnification of the boxed area in (a). b Double-positive cells are not seen in a tissue section from a 23-gw brain treated for SHH (red) followed by Olig2 staining (green). b′, b″ Higher magnification of the interventricular zone (IZ) and subplate (SP) areas. c Microglial cells (Iba1, light blue) and SHH mRNA (red) staining do not co-label cells in the 10-gw human cortex. Scale bars: a 150µm, a′ 100µm, b′ 50µm (TIF 3754 KB) [file 429_2018_1621_MOESM5_ESM.tif]

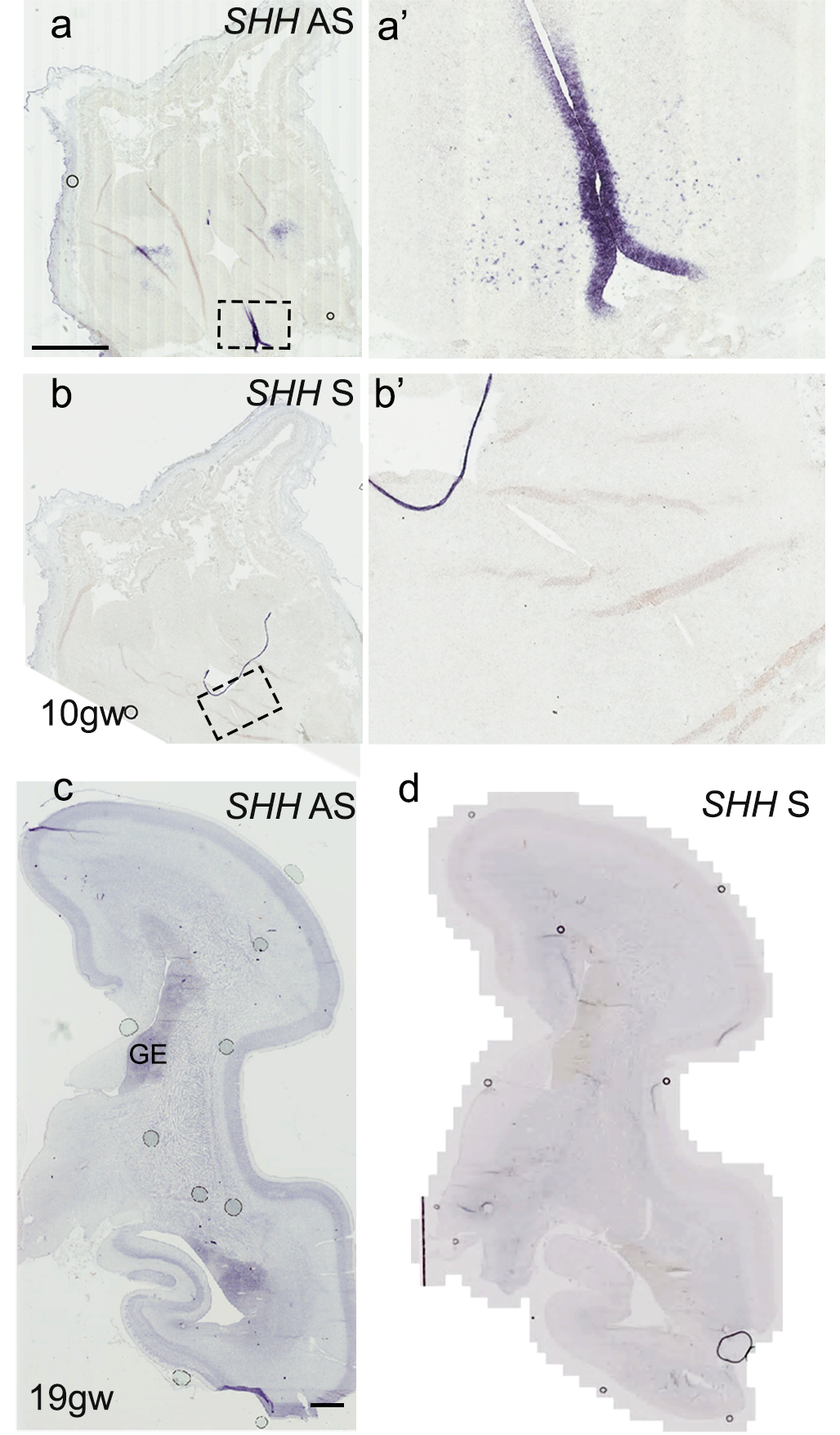

Supplement: Supplementary file 6 — Fig. S5 Sense/control in situ for SHH in 10- and 19-gw tissue. Scale bars: 2mm (TIF 9398 KB) [file 429_2018_1621_MOESM6_ESM.tif]

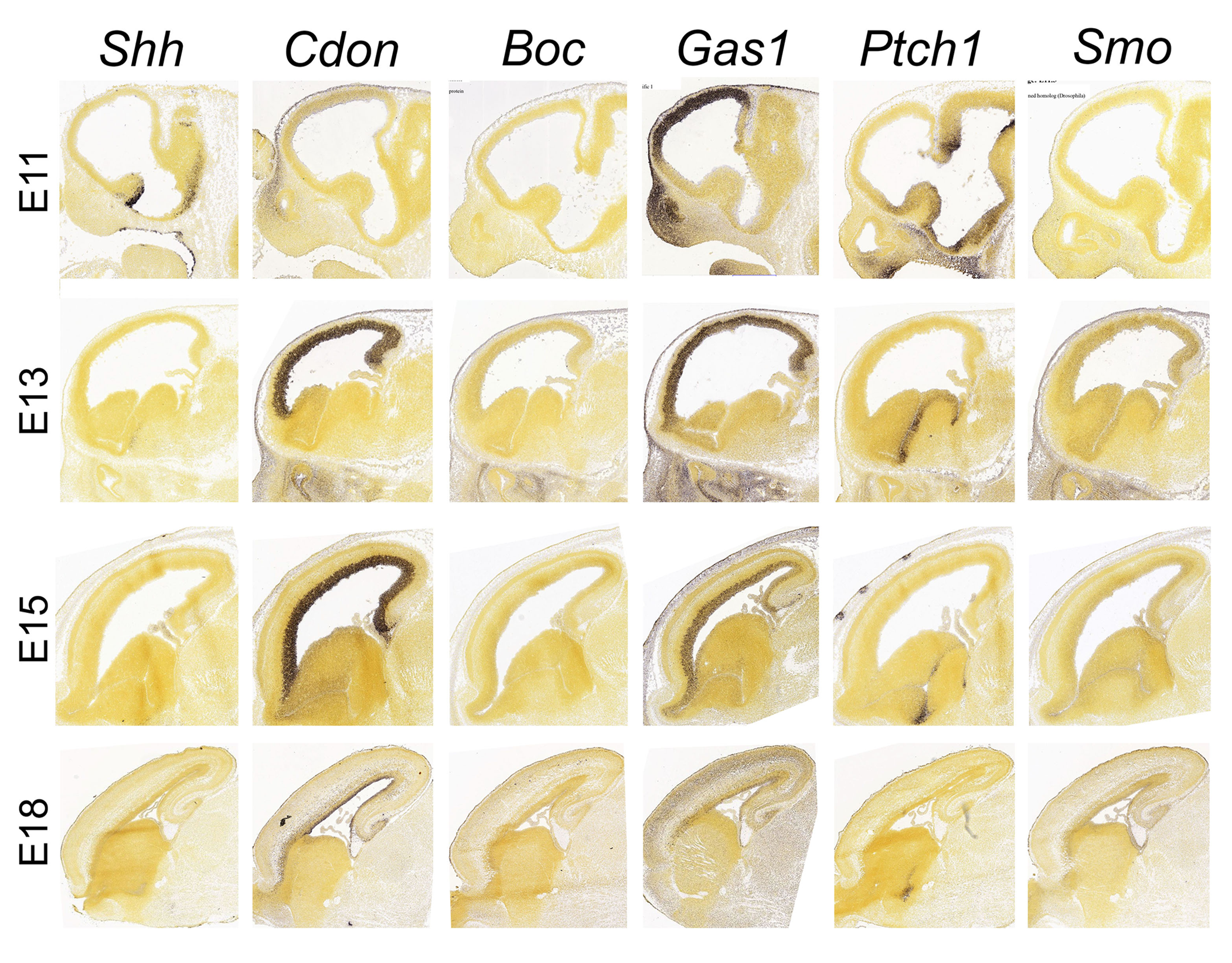

Supplement: Supplementary file 7 — Fig. S6 Expression pattern of Shh-signaling genes in the embryonic mouse brain. Data obtained from the Allen Brain Atlas (TIF 11830 KB) [file 429_2018_1621_MOESM7_ESM.tif]

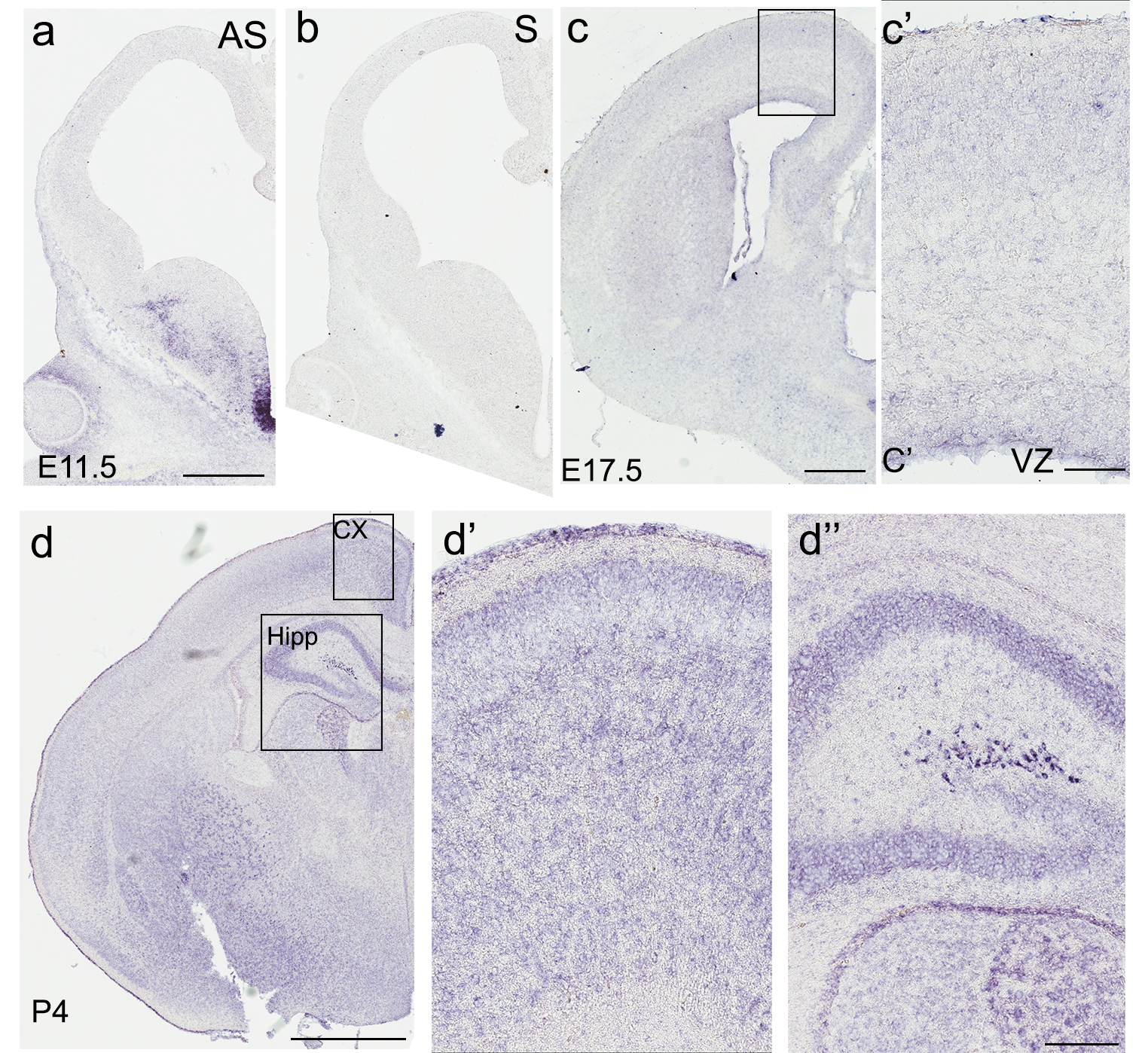

Supplement: Supplementary file 8 — Fig. S7 In situ hybridizations on mouse brain tissue with the human SHH antisense (AS) and sense (S) probe. Only (b) was probed with the sense probe. Scale bars: a 400µm, c 500µm, c’ 100µm, d 1mm, d″ 150µm (TIF 5166 KB) [file 429_2018_1621_MOESM8_ESM.tif]

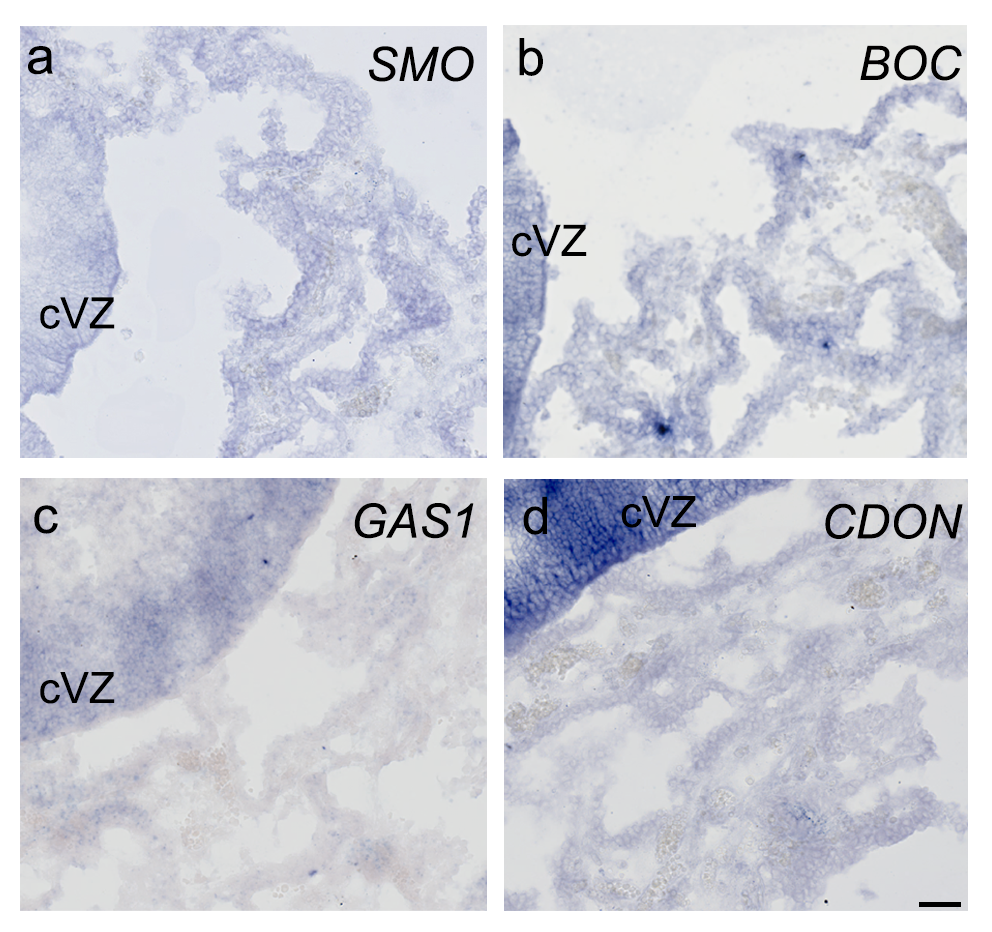

Supplement: Supplementary file 9 — Fig. S8 Expression of SMO and SHH receptors in the 10 gw Choroid Plexus. a SMO expression, b BOC, c GAS1 and d CDON. Scale bar: 50µm (TIF 5016 KB) [file 429_2018_1621_MOESM9_ESM.tif]
